# Separating obsessive-compulsive disorder from the self. A qualitative study of family member perceptions

**DOI:** 10.1186/s12888-017-1470-4

**Published:** 2017-09-07

**Authors:** Rebecca Pedley, Penny Bee, Katherine Berry, Alison Wearden

**Affiliations:** 1 Division of Psychology and Mental Health, School of Health Sciences, Faculty of Biology, Medicine and Health, Manchester Academic Health Science Centre, The University of Manchester, Zochonis Building, Oxford Road, Manchester, M13 9PL UK; 2 Division of Nursing, Midwifery and Social Work, School of Health Sciences, Faculty of Biology, Medicine and Health, Manchester Academic Health Science Centre, The University of Manchester, Jean McFarlane Building, Oxford Road, Manchester, M13 9PL UK; 3 Division of Psychology and Mental Health, School of Health Sciences & Manchester Centre for Health Psychology, Faculty of Biology, Medicine and Health, Manchester Academic Health Science Centre, The University of Manchester, Room 1.4 Coupland 1 Building, Oxford Road, Manchester, M13 9PL UK

**Keywords:** Obsessive-compulsive disorder, OCD, Illness perceptions, Beliefs

## Abstract

**Background:**

Obsessive-compulsive disorder (OCD) is a condition which can have major effects on the life of both the sufferer and their family members. Previous research has shown that the impact of illness on family members is related to their conceptualisation of the illness. In the present study we used qualitative methods to explore illness perceptions in family members of people with OCD.

**Method:**

Fourteen family members of people meeting diagnostic criteria for OCD within the previous year took part in a semi-structured interview. Transcribed interviews were analysed using thematic analysis.

**Results:**

OCD was viewed as originating from non-modifiable endogenous factors, particularly personal characteristics. Ambiguity about the boundary between OCD and the person was further heightened by a lack of distinction in family members’ interpretations about which behaviours were a problematic symptom of a mental health problem and which were behaviours performed for enjoyment or the purposeful pursuit of a goal. The perceived close relationship between OCD and the person appeared to lead to pessimism regarding the likelihood of recovery. Some individuals viewed OCD as presenting on a continuum such that individuals with sub-clinical symptoms exist on the same spectrum as those with the mental health problem. For some however, labelling of sub-clinical symptoms as OCD by members of the public was a source of frustration for families, who felt that the severity of OCD was unrecognised.

**Conclusions:**

Family members’ perceptions of the link between OCD and the person and of a spectrum of OCD presentation within the general population, may represent important dimensions of illness perception, which are not currently represented within existing models or assessment measures of illness perception. The perceptions that individuals hold about a health problem have been shown to be important in determining their coping responses to that condition. Further study using larger samples and quantitative methods are needed to understand whether these novel perceptions are associated with coping responses and outcomes in family members and people with OCD. If linked, clinicians may need to identify and challenge unhelpful family member perceptions as part of psychological therapy for families living with OCD.

## Background

Obsessive-compulsive disorder (OCD) is a debilitating mental health problem characterised by recurring, unwanted thoughts, images and impulses (obsessions) which the individual feels compelled to ameliorate with repetitive physical or mental acts, or compulsions [1]. A recent review of the literature found that people with OCD suffer from reduced quality of life compared to people in the general population [2]. In addition to the impact of OCD on the person experiencing the mental health problem, the family members who support them experience reduced quality of life [3, 4] and a significant level of burden [5], which may be comparable to burden levels experienced by relatives of people with psychotic disorders [6].

Family members can become embroiled in their relatives’ OCD symptoms, assisting them in rituals and modifying their daily routine to help the relative [7]. Whilst these behaviours may be motivated by the desire to relieve the sufferer’s distress, modern psychological treatments advocate that family members’ so-called ‘accommodation’ of OCD symptoms should in fact be curtailed during treatment [8], as these behaviours may have the inadvertent long-term consequence of reinforcing OCD symptoms, worsening symptomatology. In support of this view, a recent meta-analysis found that higher levels of family accommodation were moderately associated with increased symptom severity, though the direction of causality is unclear [9].

Despite the level of distress and disruption that OCD causes to family members, coupled with the negative impact of family members’ accommodating behaviours on the sufferer, little attention has been given to factors driving family members’ behaviours, such as their perceptions of OCD. In physical health conditions, a person’s perceptions of their own illness have been shown to be associated with coping responses and outcomes [10]. A recent systematic review provided evidence that patients’ illness perceptions were associated with a range of coping responses and outcomes in mental health conditions [11]. The authors however, noted that a limitation of the literature is the employment of quantitative measures of illness perception without first establishing whether these measures fully captured illness perceptions in mental health conditions [11]. There is a paucity of research examining illness perceptions in the family members of people with mental health problems, however in one example involving the family members of people with psychosis, illness perceptions were associated with family member responses (e.g. burden) [12].

A better understanding of family members’ illness perceptions about OCD could help to illuminate factors that lead to burden and distress in the family members themselves, as well as those perceptions which may guide their coping behaviours, such as help seeking and accommodating behaviours. The data presented here were collected in the course of a larger series of studies, which included a qualitative study, aimed at developing a questionnaire measure of family members’ perceptions of OCD. The present study aimed to explore family member perceptions of the relationship between OCD and the sufferer. It is the first study to our knowledge to employ a qualitative methodology to examine illness perceptions in the family members of people with OCD. A qualitative methodology allows for an exploration of family member views without imposing pre-determined ideas onto the data, enabling an in-depth exploration of complex ideas.

## Method

Ethical approval was granted by the NRES Committee North West – Lancaster (Ref: 13/NW/0506).

### Recruitment

Participants were recruited via two primary routes: 1) Recruitment advertisements (e.g. OCD charity websites, social media advertisements placed by anxiety charities). 2) Inviting individuals with OCD who were taking part in a UK-based multi-site randomised controlled trial (OCTET, [13, 14]) to nominate a family member. To ensure diversity in the family relation type (e.g. parent, partner) and OCD symptom sub-type, these participant characteristics were monitored as recruitment progressed. No changes to the sampling strategy were deemed necessary as representation of a variety of OCD sub-types and key relationship types (parents and partners) naturally occurred.

### Sample

Table 1 provides details of family member eligibility criteria.

**Table 1:** Eligibility criteria for participants

	Inclusion criteria	Exclusion criteria
All participants	Age 16 or above	Organic brain disease
	Able to give informed consent	Current psychosis
	English speaking	
Participants with OCD only	Meeting diagnostic criteria for OCD within the last 12 months, (module G of the Mini-International Neuropsychiatric Interview; [15])	
Family members only	Spends an average of 10 h face-to-face contact per week with the person with OCD, for at least a year	

### Procedure

Data collection took place between September 2013 and October 2015. Participants were recruited as dyads; the person with OCD and family member. All participants gave fully informed consent to participate. All Interviews were undertaken by the first author [RP]; a female PhD student with substantial experience and formal training in collecting and analysing qualitative data, particularly within mental health populations. The eligibility criteria for both participants with OCD and their family members are presented in Table 1.

#### Assessing the eligibility of participants with OCD

Family member interviews did not proceed until the person with OCD within each pair undertook a short interview where informed written consent was taken and eligibility established (see Table 1); this interview included administration of module G of the Mini-International Neuropsychiatric Interview (M.I.N.I; [15]), to confirm OCD diagnosis. The purpose of this assessment was twofold; to ensure that the family member had the explicit consent of the relative with OCD so that family members felt able to speak openly. Secondly, to ensure that family members had an eligible relative with confirmed OCD. Participants with OCD recruited via the OCTET trial undertook the same procedures although the M.I.N.I was not repeated (if taken within 12 months), as this assessment had been met at recruitment to the trial. In addition, though not forming part of the eligibility criteria, all participants with OCD completed a background information questionnaire and undertook the symptom checklist from the Yale-Brown Obsessive Compulsive Scale (Y-BOCS, [16]) in order to identify the types of OCD symptoms experienced.

#### Family member interviews

To ensure participants were able to speak about their experiences openly and in confidence, face-to-face interviews with family members took place on a one-to-one basis (in all but one case, in the absence of the person with OCD) in the person’s home or in another private location of the participant’s choice (e.g. a private room in the university). Where a face-to-face interview was not possible (e.g. due to geographical location), interviews were conducted by telephone. The semi-structured interview schedule (Table 2) comprised of eight open questions (with follow-up prompts) designed to explore how the family member understood OCD in terms of their knowledge as well as their beliefs about its development, presentation and impact. Family members were also asked about their role in managing their relatives’ OCD. The guide was developed by the research team, informed by a recent scope of the literature on family burden and further refined with the assistance of a person with lived experience of OCD. The course of the interview was flexible, allowing the interviewer to follow up with prompts or additional questions as topics arose.

**Table 2:** Interview schedule questions

Questions	Prompts
1) What do you know about OCD?	Terminology? Duration? Do you know how it should be treated? Who? i.e. are some people more vulnerable than others? Different types of OCD? Has your knowledge changed with time? How/why (e.g. meeting partner with OCD)?
2) Tell me about how your relatives’ symptoms of OCD started?	When did you recognise OCD? What led up to it? Cause? What did you do? How did you react? Looking back, were there signs before?
3) What are your relative’s symptoms like at present?	Do symptoms fluctuate? If so, what improves symptoms/worsens symptoms? E.g. treatment. Do you impact on their symptoms? Any triggers? Contrast how this week is compared to previous weeks.
4) Tell me about a typical day in your relative’s life.	How does your relative’s OCD affect you? Practically (in terms of caring, lifestyle change) & emotionally (how do they feel about it). Any positives? What about other family members? Does this fluctuate? Is this important to you?
5) Do you play any role in managing your relative’s OCD?	Has this changed with time? Why/how?
6) Tell me what the future looks like at the moment?	Stay the same, improve/worsen? Will circumstances/impact on you differ in future? Do you ever see your relative being cured?
7) Is there anything that does help/would help support you as a relative of someone with OCD?	
8) Is there anything else that you would like to add?	

Interviews lasted 27–95 min. No members of the research team had a prior relationship with any of the participants. The interviewer made brief field notes immediately after the interview, paying particular attention to any contextual factors influencing how the interview progressed, e.g. impressions of the openness of the participant. The purpose of these notes was to assist with interpretation of data during the analysis stage. Interviews were transcribed verbatim. Transcripts were not sent to participants for correction but were checked against the audio recording by the interviewer for accuracy.

As the primary purpose of data collection was to identify and characterise family members’ perceptions of OCD, data saturation was reached when no new dimensions of perception were identified.

### Analysis

Transcripts were thematically analysed using a data-driven approach. Braun and Clark [17] argue that thematic analysis can be used to provide a rich overview of the whole data set, however a second approach involves focusing in greater depth on a particular latent theme within the data. The latter approach was taken and consequently, data were coded inductively such that codes were closely related to the data as opposed to being driven by existing theory. Transcripts were shared with the research team as data collection progressed and discussed at regular team meetings, allowing the identification of emerging themes. RP read all transcripts repeatedly with reference to the field notes (which helped to ensure data were interpreted with as close recollection of the interview as possible), making notes about potential codes. RP then worked through each transcript systematically and coded with reference to the specific research aim using NVivo software [18]. Coded portions of transcripts were shared at team meetings [RP, PB, KB, AW] and discussed to develop and refine themes.

## Results

**Fig. 1:**
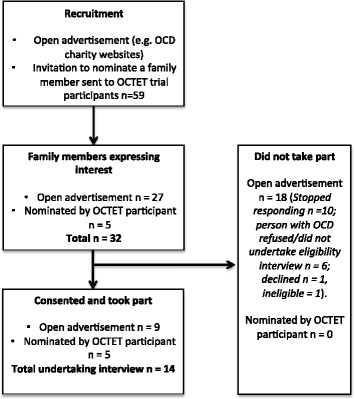
Flow of participant recruitment

Figure 1 provides details of the recruitment flow through the study.

Characteristics of the 14 family members who consented and took part in an interview can be seen in Table 3. Personal details such as age, length of relationship are reported in bands to preserve the anonymity of participants. The persons with OCD undertaking eligibility interviews belonged to the following age bands: four participants aged 16–24, one participant age 25–34, eight participants aged 35–49, and one participant aged 50–71. Participant completion of the Y-BOCS checklist indicated that the included sample experienced a wide range of symptoms, for example, obsessions and compulsions around themes of contamination, unwanted sexual thoughts, being responsible for harm, superstition and the ‘need to know’. Most participants indicated that they experienced multiple types of OCD symptoms.

**Table 3:** Characteristics of family members

ID	Relationship to person with OCD	Interview setting	Age of family member at interview (years)	Average time spent per week with person with OCD (hours)	Length of time relative has experienced OCD (years)	Length of relationship with relative experiencing OCD (years)
1	Wife	Face to face	35–49	40–49	30–39	20–29
2	Husband	Telephone	50–71	40–49	20–29	20–29
3	Husband	Face to face	25–34	50+	0–9	0–9
4	Mother	Face to face	35–49	50+	10–19	Since birth
5	Husband	Face to face	50–71	50+	10–19	0–9
6	Wife	Face to face	25–34	40–49	10–19	10–19
7	Wife	Face to face	35–49	30–39	30–39	20–29
8	Husband	Face to face	50–71	30–39	10–19	10–19
9	Husband	Phone	35–49	50+	20–29	10–19
10	Wife	Face to face	50–71	30–39	30–39	10–19
11	Wife	Phone	25–34	30–39	10–19	0–9
12	Mother	Phone	50–71	30–39	0–9	Since birth
13	Mother	Phone	35–49	20–29	0–9	Since birth
14	Mother	Phone	50–71	30–39	10–19	Since birth

Three themes were derived from the analysis: 1) The inextricable link between OCD and the self, 2) A trait-based spectrum in the general population, 3) Where personality ends and OCD begins; interpreting day-to-day behaviour in relatives.

### The inextricable link between OCD and the self

The predominant view amongst the family members was that their relative’s OCD had resulted from a predisposition to the disorder, arising from a variety of intrinsic causes, such as a ‘chemical imbalance’, genetics, or ‘brain wiring’. In these ‘predisposed’ individuals, family members perceived environmental factors (such as traumatic events, stress) as important in both triggering the initial onset of OCD, as well as activating further symptom exacerbations.

“…*it’s connected to, like, a chemical imbalance… If you’re gonna have OCD, you’re gonna have OCD. It’s not something you suddenly wake up and get, it’s something that’s within you and a stressful situation can trigger a bad episode…”* (participant 6, wife)

A prominent way of characterising relatives’ predisposition to OCD was to describe vulnerability as related to a fundamental and enduring characteristic of the individual, such as their ‘personality’ or ‘character’. The language used by family members suggested that they deemed certain ‘types’ of people to be vulnerable to OCD. These views were not necessarily mutually exclusive to biological explanations of OCD; in some cases these views could exist alongside each other. The following individual, who described OCD as a “tendency in your character”, saw his wife’s personality as biologically determined:

“*I suppose again it [having OCD] depends on your personality, just how your brain is wired.*” (participant 9, husband)

Some family members were more specific about how vulnerability to OCD was linked to their relative’s individual characteristics. One spouse, who emphasised that OCD was an ‘illness’ implicated her husband’s “obsessive mind” in causing OCD, which she likened to people who have an “addictive personality” (e.g. alcohol addiction). The spouse went further, linking vulnerability to OCD to positive aspects of her husband’s temperament:

*“…sometimes people who are very caring sensitive people, get upset by these thoughts [intrusive thoughts] because they are very caring, sensitive people and they wouldn’t want to harm anybody and that would be their worst fear…”* (participant 7, wife)

It is worth noting that although this spouse seemed to suggest that her husband’s character made him vulnerable to OCD, she regarded the content of her husband’s harm obsessions as distinct, stating “it’s different from your personality because there’s no way that my husband would ever harm anybody.” Another participant did not use the term ‘personality’ at all, but nevertheless linked vulnerability to OCD to a permanent characteristic in her son, i.e. his way of thinking. Though these parents questioned whether their parenting and tendency to want their child to “to do things well” had contributed to their son’s OCD, the factors causing vulnerability now seemed to lie so deeply within their son’s character that it seemed difficult to conceive that he could ever be free of OCD:

Participant: *“… I hope I get proved wrong, but I can’t see it being… his life ever being OCD-free. I mean, he’ll have good times and he’ll have bad times, but I don’t think it will ever totally go.”*

Interviewer: *“And why do you think that?”*

Participant: *“Just the way his mind works. The way he sees things, the way he processes things. Looking back, it’s the way he’s thought, the way he’s behaved from being younger.”* (participant 13, mother)

Similarly, other family members attributing OCD to an intrinsic cause appeared to hold the view that vulnerability to OCD would be life-long; even if OCD symptoms improved, there would always be the potential for their return under certain triggers, such as stress. Vulnerability was therefore viewed as lying inherently within the individual. The belief in a permanent link between OCD and the sufferer suggests that some family members see OCD as intrinsically linked to ‘who they are’ as a person. Indeed, in one family member’s case, the permanence of this vulnerability appeared to lead to the acceptance of OCD as part of his wife’s identity:

*“I know it’s not dealt with in a way. It’s there, it’s part of her, and she has to live with it and that’s that really.”* (participant 9, husband)

In extreme cases, the acceptance of OCD as part of the person appeared to lead to the whole family accommodating their loved one’s OCD symptoms:

“…there’s no, ‘daddy isn’t right’ or ‘daddy’s not well’ or ‘daddy just you know’, it’s just ‘daddy just needs to check that before we leave.” (participant 1, wife)

In a case which subtly differed from those described so far, OCD was seen as emerging as a consequence of a more dominant (unrelated) condition that had caused loss of confidence and self-esteem, leading to an exacerbation of the relatives’ personality ‘quirks’. Here, although the family member seemed to relate OCD symptoms to personality, OCD did not seem to be viewed as a condition in its own right requiring treatment, but instead, secondary to more severe conditions. As a consequence, this family member seemed to suggest that obsessive symptoms were not permanent:

“…*if she had the support to adequately deal with them (primary illnesses), I think all of her mental health conditions would really die down to the point where they were just within the normal range for her personality*”. (participant 3, husband)

### A trait-based spectrum in the general population

A number of the family members suggested that rather than there being a qualitative difference between those with or without a clinical diagnosis of OCD, the disorder instead existed on a spectrum of severity within the general population:

“…*everybody has a little bit of OCD. I think everybody has routines and things and habits and rituals, but the majority of people just do them without thinking about it. And if something happens that they don’t happen to fulfil it, most people would just say ‘ah never-mind, it’s alright’, but the ones who have a higher form of OCD, that’s probably not the right word, but put it on a strength to one to ten. Something more than one or two, they would struggle to say you know ‘never-mind’*…” (participant 1, wife).

A spectrum presentation of OCD meant that it was possible for people without diagnosed OCD to experience sub-clinical traits of OCD. The following participant whose son’s OCD symptoms were so severe that he was unable to remain in education, explained how, as she put it, “lots of people have mild OCD” which did not negatively affect functioning:

“…*my [other family member] checks plugs and things, and she functions, you know, she’s got a job and kids and, but she functions. And I have other friends who have some funny ways that, yeah, - OCD - mild, but they function. So, I realised it is quite common on a low level that is liveable with…*”*(*participant 12, mother)

Participants who held this view seemed to be using the term ‘OCD’ to describe behaviours in people without apparent distress and disability, suggestive of a view of OCD as a ‘trait’ that could appear to varying degrees, as opposed to an illness dichotomy that is either present or absent. Contrary to this view, other participants expressed frustration about the recent popularisation (e.g. in the media) of using the ‘OCD’ label to describe any meticulous or ritualistic behaviour in a person without diagnosed OCD. These participants believed that this misuse could result in the trivialisation of a debilitating mental health problem. Here, a more dichotomous view of illness and wellness was emphasised, such that ‘OCD’ should only be applied to behaviour that causes dysfunction:

“…*people say they’ve got OCD because they’re very fussy and particular and they like their books in order and things like that. But I think that trivialises it because a lot of people say ‘I’m very OCD about this, I’m OCD about that’ but that’s not an illness, I think it’s only an illness when it becomes debilitating*.” (participant 7, wife)

One family member who endorsed a spectrum view of OCD from “mild to severe”, suggesting that we ‘all’ have a “very mild” form of OCD, then appeared to criticise similar use of the ‘OCD’ label by others to describe behaviour in someone without the disorder stating:

“…*people say, “I’m dead OCD about that”, and I think, “Well, you don’t know what you’re talking about”. They mean they’re a bit keen on cleaning something or making something nice. That’s not OCD at all. That’s just being particular about something. If you have real OCD it’s very, very debilitating.”* (participant 10, wife).

The coexistence of these views in this family member brings into question whether those disagreeing with a more liberal use of the OCD label necessarily disagree with those expressing a spectrum-based presentation of OCD. Whilst the former group appeared to be discussing ‘traits’, the second group emphasised the threshold for a ‘disorder’, i.e. the point where symptoms become more than ‘traits’ and cause disability and distress. The frustration of family members objecting to the application of the OCD label to non-clinical behaviour appeared to link to a perceived lack of public understanding of the high level of burden and distress that OCD causes to sufferers and their families. When asked what support would be beneficial for her family, one mother seemed to indicate that a failure of other people to appreciate the severity of OCD was in itself a difficulty in her experience of coping with OCD:

“…*people understanding it a little better. I get infuriated when people say ‘oh I’ve got OCD’. I’m thinking ‘no you haven’t, you’ve just a funny little ritual. People’s understanding of it would help. I work in [work-place] and people who should know better, calling people ‘nutters’ and ‘weird…”* (participant 4, mother)

### Where personality ends and OCD begins; interpreting day-to-day behaviour in relatives

In the previous section, it was suggested that some family members do not perceive a clear distinction between OCD symptoms and ‘sub-clinical’ behaviour. When family members talked about their own relative with OCD, there was a lack of clarity about which aspects of their thinking and behaviour were driven by OCD. In the earlier stages of their experience of supporting the person with OCD, some family members struggled to recognise symptoms. A spouse explained that despite meeting her husband after the onset of his symptoms, it was only upon noticing the fluctuating course of her husband’s OCD symptoms that she began to separate OCD from his personality:

“*That’s the way he’d always been, ever since I’ve known him. So I just thought that was part of his personality. I just thought that was him. So it’s only getting to know him over the years, that I realised that it does get better and get worse*.” (participant 11, Wife)

Behaviours that appeared to have a useful or positive outcome could be interpreted to be non-problematic and therefore part of a person’s ‘usual’ behaviour. There was some evidence that this could lead to a delay in recognising that the person was experiencing distress. One mother described how her son had later confessed (after being diagnosed with OCD) that his highly productive behaviours had in fact been driven by an obsession, which he had been unable to control:

“…*He was brilliantly artistic, read endlessly, always reading, always self-motivated to learn, which he said was an obsession. He said, “Mum, I did it all obsessively though, I couldn’t stop*”…*.”* (participant 12, mother).

The mother became aware that her son was experiencing distress when he later spoke of his contamination obsessions, which immediately prompted her to seek treatment for her son. There was some evidence that behaviours which appeared ‘positive’ were continued to be viewed more favourably than other OCD symptoms once a diagnosis was established. The following participant suggested that her son’s obsessional thinking and behaviour, which for the most part caused him great distress, could occasionally be used to his advantage:

“… *we do joke about good obsessions and bad obsessions and he does take it in the vein that it’s meant. Like when he was training for his bike ride, “[Son’s name], this is one of your good obsessions”, “Yeah, I know, it’s good isn’t it?” So he can… he can see what we’re saying and something that’s a good to obsess about, and I’m not saying it’s good to obsess about anything, but I’m just saying that it’s better to obsess about that than something that’s going to make you feel worried about everything*.” (participant 13, mother)

Another spouse explained that although she would challenge symptoms that she perceived as “unreasonable”, behaviours that caused few consequences for her and her husband were tolerated:

“…*he has to know every minute detail about things. But that’s also, that’s equally something that he enjoys. It’s not something that he finds distressing. It’s only things that he finds distressing and perhaps take over, take more time…more of his time up, that I think needs to be challenged, just for his long term happiness. But little things like listening to a song over and over again, or watching TV programmes over and over again, I don’t think that’s a negative thing necessarily*.” (participant 11, wife)

Whilst it is not possible to tell whether the spouse did in fact experience no distress in this case, views such as this suggest that some family members perceive OCD as presenting on a trait-based spectrum within the sufferer. As such, OCD can drive both unwanted and distressing symptoms but also some positive, sub-clinical level manifestations that bring about limited distress or even beneficial outcomes.

In the cases discussed so far, family members seemed to attribute behaviours to OCD even if they perceived them to have little negative impact. In a small number of cases, family members questioned whether demands put on them to assist their relative could truly be attributed to OCD. In one case, this was expressed out of concern for the relative; the partner questioned whether the OCD label could cause the person to think “it’s not me, it’s the OCD making me think this”, which could lead to the person not challenging their symptoms. In another case, a spouse seemed to find the lack of distinction between her husband’s OCD symptoms from his personality difficult, stating: “I’m not sure where the OCD ends and my husband’s personality comes in.” Her description suggests that despite having doubts, she continued to accommodate her husband for the sake of their relationship:

“…*obviously it’s not his fault, so I make allowances for that. Erm, I think that’s a problem that, because we’d end up having a lot more rows if I thought it was just him being difficult. So I don’t know what he’s getting away with, what he’s not getting away with, if you like*.” (participant 10, wife)

This spouse, who was engaged in a particularly high level of accommodation compared to other participants, notably differed from the rest of the sample in that her inability to distinguish OCD from her husband’s personality appeared in itself, a source of distress.

## Discussion

The findings of this study suggest that family members perceive OCD as intertwined with the personal characteristics and identity of their loved one. Family members saw vulnerability to OCD as derived from permanent and intrinsic factors which could be biological in nature (e.g. ‘chemical imbalance’), but could also link to the individual’s own personal characteristics. As such, vulnerability to OCD was inextricably linked to the person with OCD, leading to pessimism about the likelihood of the disorder fully remitting. In some cases, family members appeared to interpret the permanence of OCD and its link to the person as a cue to accept the ‘illness’ as ‘part’ of the person’s identity. There was some evidence that such acceptance could lead to a culture within families where members accommodated symptoms due to the perception that it is a ‘normal’ part of that person’s behaviour, which they should make allowances for. Family member beliefs that lead to acceptance of OCD may be an opportunity for intervention within psychological therapies. In a recent survey based study exploring differences in perception between ethnic minority groups in the UK general population, ‘personality or emotional struggles’ was endorsed as a top 3 ‘cause’ of OCD by parents from all ethnic groups who read a case vignette describing a child with OCD [19]. Though these parents did not have a child with OCD, this suggests that linking OCD’s origin to personality may be a belief that is present even before family members come into close contact with OCD. Further exploration is however required to test whether such beliefs do indeed lead to responses, such as accommodation, in family members.

At a population level, some family members discussed their belief in OCD presenting in a spectrum across the population, whereby some individuals could exhibit sub-clinical levels of OCD. Other family members did not raise this possibility and indeed some individuals emphasised that ‘real’ OCD involves significant distress and disability. That mental health conditions exist on a spectrum of experience is an idea with some support. For example, evidence from a recent study testing the structure of psychosis in a general population sample concluded that their results “support the idea of a psychosis continuum, as it appears that psychotic symptoms cluster together in similar patterns at both clinical and subclinical levels” ([20], p. 139). That there is no distinct cut-off between mental ill health and ‘wellness’ is a view advocated by some researchers who oppose what they regard as a harmful traditional medical model, which brings about negative consequences for those diagnosed with mental health problems [21]. It is therefore possible that emphasising the similarities rather than the differences between OCD sufferers and other members of the general population may help ‘normalise’ OCD, helping family members to cope. Some family members appeared to emphasise a more dichotomous view of OCD by describing their frustrations about the failure of others to understand the severity of the mental health problem. Here, emphasising the similarities between OCD sufferers and the general population appeared to be a source of distress, rather than a ‘normalising’ coping mechanism. These findings suggest that the question as to whether or not OCD is seen as a spectrum disorder may not be of particular relevance, rather it is the perception of the general public’s understanding of the severity of the symptoms that is most important to family members. Whilst further research is needed to support or refute this possibility, findings from this study suggest that OCD public awareness campaigns may need to be sensitive to both perspectives, as each may support family members’ coping in different individuals.

Some participants discussed failing to notice symptoms initially, assuming the behaviours were due to personality. This indicates that there can be a failure to recognise distress in the person with OCD, particularly when they perceive the behaviour has produced a useful outcome; such as academic achievement. This failure of recognition could delay help seeking for that individual. That family members failed to identify OCD due to the attribution of that individual’s behaviour to personality, was a finding echoed in a qualitative study aiming to explore family member coping strategies in OCD [22]. When an OCD diagnosis had been established, family members seemed to include behaviours that were less problematic or even enjoyable as part of the mental health problem. This is contrary to the ‘diagnostic features’ specified in DSM-5, which specify that obsessions are not pleasurable or voluntary but instead experienced as “intrusive and unwanted and cause marked distress or anxiety in most individuals” [1, p 238]. DSM-5 further clarifies that compulsions are not “done for pleasure” [1]. There are two possible interpretations of these findings. First, although family members perceived certain behaviours as enjoyable or less problematic for the person with OCD, it might be the case that their distress is in fact concealed. This may lead to family members accommodating behaviours that may actually be adversely affecting the individual. Secondly, it may be that family members over-extend the diagnosis to behaviours that are not part of a mental health problem. The latter possibility may lead to a view of the individual as simply “obsessive”, leading to pessimism that such behaviour could ever be modified through treatment.

This study has a number of strengths and weaknesses. Use of a qualitative methodology has led to the identification of a number of novel illness perceptions, in particular the perception of OCD presenting on a spectrum within the population and the difficulty in distinguishing the condition from the person. These perceptions are not currently identified within current illness perception models or assessment measures, the most widely used of which are the common sense model of self-regulation [23] and the Illness Perceptions Questionnaire (IPQ-R; [24, 25]) respectively. It should be noted that ‘my personality’ is listed as a potential cause of a condition within the IPQ-R, indicating that this perception might also by reflected in understandings of physical health conditions. Quantitative study whereby these novel perceptions are measured and outcomes assessed over time (i.e. a longitudinal design) are now needed to determine whether the novel illness perceptions lead to coping responses and outcomes in family members and people with OCD. A limitation of this study is its focus on a specific question about family perceptions of OCD. Further qualitative examination is required to understand the full range of family members’ illness perceptions.

Whilst the requirement for the person with OCD to consent prior to their family member interview may have helped to foster openness in the family member interviews, it did prevent recruitment of family members whose relative with OCD declined to participate. It is possible that those individuals refusing consent may be different than those who consented, for example those refusing may have more severe OCD or more problematic family relations. This could potentially have resulted in a biased sample. Though we were aware of these limitations at the outset of the study, the first author conducting the interviews felt that it would have been difficult to proceed with interviews, without the full knowledge and consent of the person with OCD. Additionally, involvement of the person with OCD allowed us to verify diagnosis of OCD; a strength of this study. The recruitment of participants using a combination of approaches, including online recruitment (e.g. via social media) and recruitment through a multi-site treatment trial enabled individuals to be recruited from a wide geographical spread with differing experiences, a further strength of the study. Whilst family members engaged in researching OCD online (and consequently noticing the study advert) may represent a particularly motivated group of family members who may hold particular views about OCD, this was balanced by the inclusion of family members who had been invited by the person with OCD as a result of taking part in the treatment trial. In the latter case, these family members were not necessarily seeking support at the time of interview and had often engaged in few conversations with any professional about their experience of OCD. Indeed, some family members interviewed spontaneously expressed that they had benefitted from the opportunity to talk about their experiences.

It is possible that some family members would have been included in family therapies as part of any treatment delivered to their relative with OCD (e.g. psychoeducation), which could have affected their knowledge of OCD and consequently their illness perceptions. Unfortunately, we did not ask family members if they had been involved in therapy to see if previous treatment experiences did impact on perceptions; future studies should inquire as to family members’ inclusion in treatment. A final limitation of the study is the inclusion of family members who were mothers and partners/spouses. Though efforts were made to recruit participants of different relationship types, we found that spouses and mothers were most willing to participate. Future studies may benefit from aiming to recruit other relationship types, especially fathers, as these individuals may hold differing perspectives.

## Conclusions

OCD did not appear to be regarded by family members of people with the mental health problem as truly distinct or separable from the person experiencing the condition. It is possible that the blurred boundary may contribute to family member coping strategies which affect outcomes for both the family member and person with OCD, for example, by accommodating the individual’s rituals and behaviours. In addition, this study highlights that public understanding of the significant impact of OCD is important to family members, which may impact on their distress levels.

Further research using a quantitative methodology is now needed to explore whether the illness perceptions identified here lead to particular responses and outcomes in families living with OCD. If such a link is supported, family members’ beliefs about OCD may provide an important opportunity for intervention within psychological therapies, for example, by using cognitive behavioural therapy to examine the helpfulness of beliefs and the evidence that supports them. Through challenging beliefs that have a negative impact on coping and distress, such interventions could lead to benefits for both parties. Finally, the perceptions of family members discussed here emerged spontaneously from family member discourse without prompting; indicating the importance of the issue in this group.

## Abbreviations

M.I.N.I: Mini International neuropsychiatric interview
OCD: Obsessive-compulsive disorder
Y-BOCS: Yale-brown obsessive compulsive scale
